# Transformative practice and its interactional challenges in COVID-19 telephone contact tracing in Flanders

**DOI:** 10.3389/fpsyg.2023.1203897

**Published:** 2023-08-30

**Authors:** Stef Slembrouck, Mieke Vandenbroucke, Romeo De Timmerman, Anne-Sophie Bafort, Sofie Van de Geuchte

**Affiliations:** ^1^Department of Linguistics, Ghent University, Ghent, Belgium; ^2^Department of Linguistics, University of Antwerp, Antwerp, Belgium

**Keywords:** transformative sequence, telephone contact tracing, COVID-19, knowledge status clients, upgrading/downgrading strategy, use of humor, categorization work, emotional work engagement

## Abstract

This article focuses on transformative interactional practice in COVID-19 contact tracing telephone calls in Flanders (Belgium). It is based on a large corpus of recorded telephone conversations conducted by COVID-19 contact tracers with index patients in the period mid-2020 to mid-2022. The calls were conducted through government-contracted commercial call centers. For nearly 2 years and applied country-wide, this was the most prominent strategy in Belgium for breaking transmission chains. COVID-19 telephone contact tracing with infected patients counts as transformative professional work in two ways. First, in addition to the registration of recent contacts in a relevant time window, the work is oriented to awareness-raising about how patients and their co-dwellers can and should adjust their behavior by attending actively to critical aspects of the pandemic during an individual period of (potential) infection. This is the terrain of advice, interdictions and recommendations about quarantine, isolation, personal hygiene, etc. In addition, the focus on interactional attention indexes patients’ affect and emotions (e.g., anxiety, worry, or anger) in a period of health uncertainty and social isolation. The transformative work thus depends on successfully established rapport and empathetic, responsive behavior. Our analysis of the recorded conversational sequences focuses on the complexities of client-sensitive and responsive transformative sequences and highlights the constraints and affordances which surround the interactional task of ‘instructional awareness raising’ which is central to telephone contact tracing. Specifically, we detail the following dimensions of transformative sequences: (i) how do contact tracers deal with the knowledge status of clients, (ii) their use of upgrading/downgrading formulations, (iii) the use of humor and other mitigating strategies, and (iv) how contact tracers attend to interactional displays of affect and emotion. In a final section, we tie together our observations about the communication of particularized advice in a context of general measures through the twin notions of categorization/particularization-work. The findings in this paper are limited to the first step in the chain of contact tracing, i.e., telephone calls with tested and infected citizens.

## 1. Introduction

According to the World Health Organisation, contact tracing can be defined as a public health practice for identifying, assessing, and monitoring individuals who have been exposed to an infectious disease, so as to prevent its further spread amongst a community or population.^1^ Alongside mass testing and, if available, vaccination, contact tracing is undoubtedly a crucial practice to contain infectious disease outbreaks. Dar et al. (2020, p. 2) remind us how “containment is a primary roadmap to quickly halt an outbreak, which may become an epidemic and then in the worst case, turn into a pandemic, which is exactly what happened in the case of COVID-19.” Unlike symptom-based detection, contact tracing is preventive; its success ultimately depends on how fast the contacts of index patients are traced and quarantined (Juneau et al., 2020). Index patients are individuals who have been tested and diagnosed as infected; the term *index* signals that the information which they provide *points to* other individuals who need to be contacted, because they are at risk, need to quarantine, etc. At the same time, the practice of contact tracing allows “individuals (…) to relieve distress from a community’s containment measures,^2^ as it gives the infected individuals a chance to quarantine themselves voluntarily.” Contact tracing can also be expected “to increase sensitivity (…) followed by readiness for an emerging pandemic” (Dar et al., 2020, p. 2).

Typically, contact tracing combines interview-based techniques and tracing technology to identify the recent contacts of an individual who has tested positive for a disease, to evaluate the contacts’ risk of infection due to exposure, and to monitor their health and possible illness. The latter function, even though it is prevalent in contact tracing practice, is poorly captured by the label *contact tracing*, which primarily suggests data collection, processing and alerting, more than an individually tailored interactional engagement with an infected index patient. Contact tracing has been adopted as a public health practice since the 19th century. It has been used for the containment of syphilis, tuberculosis, measles, smallpox, HIV/AIDS and Ebola (Gyselen, 1994; Samoff et al., 2007; Lin et al., 2012; Greiner et al., 2015; Mbvinjo et al., 2021). It was adopted on a large scale around the world during the recent outbreak of COVID-19. Given its critical significance for the quick and efficient identification and isolation of (potential) new cases, it was intended to enable early containment and intervention and ultimately reduce the further transmission of Covid-19 by temporarily intervening in the lives of affected individuals. Mapping aspects of contact tracing’s history, Brandt (2022) notes an important shift in contact tracing from a public health approach of surveillance to one which emphasizes community engagement and support by “centering attention on informing individuals of their infections; educating them on best practices to avoid transmission; assuring that they had resources to isolate; and providing social support” (2022: 1099).

Before we continue our discussion of telephone contact tracing in terms of the transformative purpose that can thus be identified, we first provide a brief overview of the different types of contact tracing that were adopted in Flanders, Belgium during the COVID-19 pandemic. In doing so, we particularly want to highlight the sense of novelty which accompanied their introduction for the population at large. Unlike earlier practice in contexts of HIV/AIDS and tuberculosis, in the case of COVID-19, contact tracing had relevance for everyone across the country and it became a major long-term topic in news coverage (Bafort et al., 2023).

## 2. COVID-19 contact tracing in Flanders

One of the most immediate actions taken by the Belgian government during the first months of the COVID-19 outbreak in the Spring of 2020 was the roll-out of a three-tier contact tracing system to break transmission chains. Contact tracing enabled the government to document citizens who had tested positive for COVID-19, as well as the individuals who they had recently been in (close) contact with and who needed to be alerted about their exposure and possible infection. In line with WHO-recommendations and *the European Centre for Disease Prevention and Control*, this system involved two types of personalized contact tracing, with differing degrees of anonymity. Their implementation involved multiple levels of governance in the Belgian federal state (see Slembrouck, 2023 on COVID-19 as a multi-scalar engagement).

The first type was the development of automated contact tracing through a digital proximity tracing app. A smartphone app, *Coronalert*, was developed by a consortium of experts and scientists for the federal government and rolled out at a national scale (Jacob and Lawarée, 2021). At its launch in September 2020, the app was believed to be an important supportive factor in the fight against COVID-19 as “[s]uch a technological solution allows to track, in real-time, a massive number of (potentially) infected individuals within a given population (…) to isolate cases of COVID-19 and reduce the basic reproduction number (…)” (Jacob & Lawarée, 2021, p. 45). *Coronalert*, when it is installed on two smartphones, registers a cell phone carrier’s proximity to other users, and this allows, when infection occurs on either side, the tracing and alerting of contacts, without having to rely on the smart phone users’ memory or their awareness of and familiarity with specific individuals who were at some point in their vicinity. The alert app offers an anonymous form of contact tracing, as the app tracks individual proximity *via* Bluetooth. Upon infection, people (unknown to the infected individual) are alerted of the duration and distance of exposure to the virus-transmitting body (Proesmans et al., 2022). Despite its usefulness to alert people who may be strangers to one another or who were unaware of others’ proximity, this type of COVID-19 contact tracing was criticized as an instrument of mass surveillance. Low adoption rates by end-users were especially noted as detrimental to its effectiveness, as the success of automated contact tracing depends on high population uptake (Raus et al., 2021; Vogt et al., 2022; Bafort et al., 2023). Braithwaite et al. (2020, p. e607) conclude that “large-scale *manual* contact tracing is therefore still key in most contexts” (our emphasis).

The second type of contact tracing was more traditional and drew on pre-existing models. The tracing is done in the form of one-on-one interviews with index patients, with follow-up phone calls to their recent contacts (Barrat et al., 2021). In Belgium, this type occurred during the pandemic in two formats: (i) as locally-organized initiatives taken by general practitioners, health care and community workers, and (ii) as regionally-organized call center operations which were mandated by the official health agencies. The first form was anchored in several (sub)urban contexts and was highly variable in its practical organization: general practitioners engaged in contact tracing during frontline consultations with patients; local authorities set up a system of phone calls; home visits were conducted by field agents; etc. In contrast with this, the second form, organized through call centers, was applied consistently in each of the Belgian regions (Proesmans et al., 2022, p. 2).

In this paper, we specifically focus on regionally-organized telephone contact tracing in Flanders. This task was mandated by the federal and Flemish governments to a consortium of commercially run call centers, national health organizations and a consultancy firm. During the initial lockdown of early 2020, the call centers expanded their workforce of helpline operators and trained a large group of telephone contact tracers. They were not required to have any (para)medical training or professional medical background. Most lacked experience with contact tracing. The call centers recruited to a large extent amongst workers with experience in the service and communication industry who had become unemployed due to the COVID-19 lockdown (e.g., flight attendants, hotel and catering staff, stage directors, actors, etc.). Recruited workers received on the job training which covered the use of the technology, the contact tracing script as well as communication skills.

**Figure 1:**
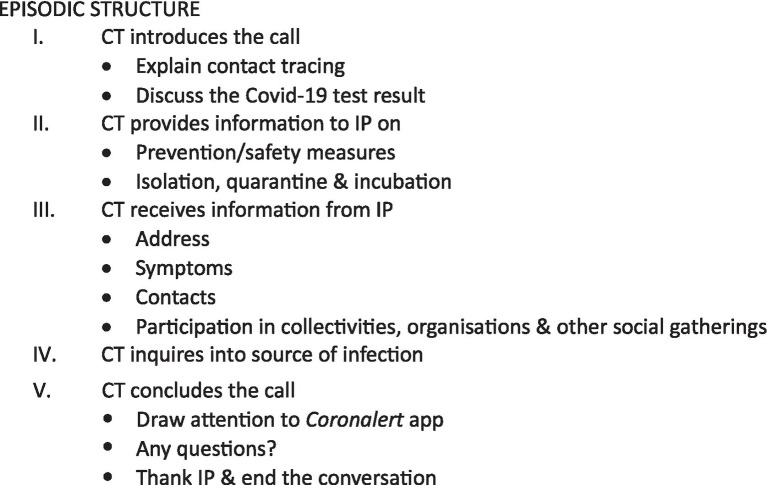
Episodic structure of the CT call.

Call center contact tracing made use of a centralized IT platform in which index patients were listed as case files in a call queue. Following entry into the system, a contact tracer would call the index patient within the next 24 hours and ask about the nature and identity of recent contacts in the relevant, infection-prone time window. After this initial call, the reported contacts received secondary calls by contact tracers notifying them of the risk and possible exposure, and when applicable, the outcome of these secondary calls led to subsequent testing and measures of quarantine. The case file for each index patient on the platform contained a long list of sections with categories and items of information for which the index patient’s answers were recorded as well as a list of categories and items to be communicated. This list functioned as a script for the contact tracer to conduct their interviews. Previous research on this type of Covid-19 contact tracing call center conversation (De Timmerman et al., 2023) has documented how the script informed the episodic structure for the encounter. As detailed in Figure 1 below, two major stages of information exchange can be discerned: one in which the contact tracer provides information and instructions on prevention/safety measures as well as explaining isolation, quarantine and incubation periods (section II in Figure 1) and one in which the index patient provides relevant information about recent contacts, alongside information about infection and symptoms (sections III and IV). The script also promoted the use of the *Coronalert* app (section V).

The research reported in this paper concentrates on episodes II and III. It is limited to telephone calls with index patients.

### 2.1. Telephone contact tracing as interactional work in an evolving context

COVID-19 telephone contact tracing can be understood in terms of goal-oriented interactional work in an evolving institutional context of public health management. First, it is oriented to awareness-raising about how patients and their co-dwellers can and should attend to various aspects of the pandemic during a critical, individual period of actual/potential infection. This is the instrumental terrain of advice, interdictions and recommendations about a range of relevant categories: quarantine, isolation, personal hygiene, preparation of food, and so on. This stage of the call is mostly centered around directing behavior and rendering it instrumental to containing the disease (e.g., wear a mouth mask while you are in the room with others in the house; the infected person uses a separate toilet, if available; etc.). The purpose here is to secure safe conduct in the house and outside (items raised include shopping, taking the dog for a walk, etc.). Secondly, the contact tracer’s focus lies on the interactional management of a particular relationship with the index patient, one which preferably not only guarantees successful uptake of instruction and advice, but also attends to the potential challenges and pitfalls inherent in the task of having to inform and instruct your interlocutor. In part, the challenges stem from the contact tracer’s need to impose on others’ freedom to act by performing a number of face threatening acts (Brown and Levinson, 1987), especially ‘representatives’ (to inform and remind people of rules, measures and state of affairs which apply) and ‘directives’ (to tell people what to do and what not). On top of its largely behavior-constraining orientation to prohibition and prevention, a major difference with telephone helpline interactions (Bloch and Leydon 2019) must be noted: the index patient is not actively seeking help. Instead, the contact tracing call is initiated by the institution. Arguably, this adds to the odds against the contact tracer: their callers at the other end of the line may already be well-informed *via* others; they may be quite ill at the time of the phone call; they may be annoyed by the un announced intrusion into their private lives; they may be distressed or anxious upon receiving the news of a positive test result and reluctant to interact; etc. In the specific case of COVID-19, the contact tracing calls had to be done in a field of social practice which was imbued with a heightened sense of personal and collective risk, a context of distress, uncertainty and quickly evolving circumstances. As noted earlier, the Flemish telephone contact tracers were instructed to adopt an empathetic and supportive stance during their phone calls. Attention to index patients’ voiced concerns, moments of panic and distress, emotional responses, etc. was part and parcel of this. As a result, doing contact tracing in the period 2020–2022 meant that the talk was often also about the current stage of the pandemic, the introduction of ‘new’ measures (incl. tightened measures, as well as relaxed ones and suspensions; the likely development of vaccines; the timing of their availability; risks attached to vaccination, etc.).

Given the unfamiliar nature of COVID-19, especially in the early stages of the pandemic, the interactional challenges for the contact tracer may have appeared huge. In this respect, it is important to attend to the state of play at the point in time the cited interactions were recorded.^3^ While telephone contact tracing in Flanders started in May 2020, the recorded data corpus on which this article is based is mostly situated in the period December 2020–January 2021, by which time Flanders had gone through two periods of lockdown, the one in the Spring of 2020 stricter than the second lockdown which followed in the Autumn of 2022 (the Summer of 2021 was characterized by a partial/temporary relaxation of the measures). It is best to assume that by the time the data was collected, a lot of information about how to quarantine and self-isolate, about hand hygiene and mask wearing, etc. was already well-established in the public mind. At that time, the country was also gearing up for an expected start of vaccination in the Spring of 2021.

## 3. Contact tracing as transformative work

Contact tracing talk can be understood as transformative work. This invites analytical attention to transformative sequences of talk. Transformative work pertains particularly to the sections where index patients are given advice and instructed what to do. Peräkylä (2019) observes with reference to psychotherapy that sequentially accomplished contributions to talk and interaction can enable a process of transformation of experience, which pertains to the referents talked about, the client’s emotions and the momentary relations which occur between therapist and client. This highlights how professional intervention work through interaction with clients is oriented to awareness transformation in the client and how the face-to-face rapport between client and professional is vitally attended to as part of this. Following Peräkylä (2019) and others (e.g., Muntigl et al., 2017; Knol et al., 2020), sequential analysis of talk has the capacity to show how interaction unfolds in the service of institutional and professional tasks; it can also show how processes of cognitive and affective change take place as part of that. In short, detailed interactional analysis can demonstrate how an intervention took place and show attempted transformation at work by detailing how transformative sequences of talk are organized.

Important qualifications must be added when we situate the work of the Flemish contact tracers among the various forms of transformative work that one may come across in different professional and occupational contexts.

(i) Unlike the social worker or the psychotherapist, the (Flemish) telephone contact tracer was not a qualified professional. The contact tracers were occupational workers, who received training on the job, with no specific (medical, paramedical or psychological) pre-qualification being required.

(ii) In contrast with work done over the span of successive face-to-face engagements (as is mostly the case in social work or therapy), the tracer’s contact with the index patient consisted of just one phone call with a strategic timing (the index patient had just been diagnosed as infected by COVID-19). Nor was the call scheduled at a time of convenience for the patient. The brevity of the intervention, the one-off nature of the phone calls and their possible unexpectedness have implications for the scope of the work that can be accomplished. How much can be accomplished in a short telephone call with a client you have not talked to or met earlier, and whom you know virtually nothing about before dialing their number? At the same time, the contact was neither *in situ*, nor face-to-face, but instead: over the phone, with interlocutors who do not share a visual field of perception.

(iii) Thirdly, the question must be raised about the specific focus on the transformation which was envisaged in the contact tracing call. We prefer to characterize contact tracing as oriented primarily to instrumental transformative work which ideally brings about clarity and decisiveness about how to act and behave during a critical but short period of time following infection. The prevailing instrumental orientation of the contact tracing work was also informed by considerations that acting low key but doing so decisively offered the best chances at a successful accomplishment of a set of preferred client behaviors. Nevertheless, the tracers were briefed to embrace a (frontline medical) model of interaction rooted in empathetic response and patient-centeredness, in which the caller can also determine what is being talked about. Emotion, affect and a certain degree of topical leeway were viewed as part and parcel of the occupational brief (Bafort et al., 2023). While affect undoubtedly fundamentally frames an interaction in contexts of therapy, in practice, this turns out much less the case in contact tracing.

Transformative work is also associated with a pivotal intervention. Discussed at length in Noakes (2014, p. 179), who writes on social work in the field of criminal justice, the author defines transformative work more loosely, while stressing timing and assistance: “the social worker essentially advises the client about the possibility of change for the client and looks at this opportunity to assist the client with […] a transformative process. […] the social worker might tell the client they are ‘at a fork in the road’ and remind the client that it is an opportunity to possibly take a different path.” Similarly, the COVID-19 contact tracing call, as it is occasioned by the infection diagnosis, comes at a critical moment of tightened restrictions on and instructions for how to behave, doing so in the interest of people around the infected persons, at their home and beyond. Needless to add, formulations of risk and moral responsibility are never far away [see De Timmerman et al. (2023) for an analysis of contact tracing calls in relation to the construction of risk and responsibility].

(iv) Finally, in our analysis, we do not wish to make any claims about transformation being successfully accomplished, other than pointing out how particular responses may hint at (un)successful uptake. Our mainstay is with the analysis of transformative interactional sequences.

## 4. Data materials and methods

This article draws on a corpus of 220 contact tracing calls which were collected over a period of 14 months (from December 2020 until February 2022) within the context of a one-year inter-university research project funded by the Flemish Research Council (FWO).^4^ The project was carried out by a transdisciplinary team of (socio)linguists, epidemiologists, medical experts, sociologists and moral scientists; the team included a representative of the Flemish Agency of Health and Care and one of the private call center companies that employed contact tracers. The primary focus of the project was an interactional map of current contact tracing practice, and to develop empirically based recommendations which could be implemented through in-service coaching and updated recruitment procedures (*cf.* Hepburn et al., 2014, p. 252).

To carry out this twofold research agenda, the project was divided into three distinct phases. In a first phase, we collected 100 contact tracing calls (in Dutch) between contact tracers and index patients. Based on our analysis of the phase 1 corpus, we formulated a number of practical recommendations which were implemented in a training module for a small number of contact tracers. We subsequently recorded 70 Dutch calls with a control group and a pilot group to measure the impact of our recommendations and training on actual practice, while registering evolutions in metapragmatic awareness (pre-and post-measurements). In a third and final phase of the project, we recorded 50 contact tracing calls in languages other than Dutch. This sub-corpus features interactions in English, French, Arabic and Turkish. All recorded interactions hinged on written consent from the contact tracers, and two-fold oral consent from the index patients, which was obtained before and confirmed after the contact tracing call.

To analyze the interactional data across the project’s three phases, a combination of interactional sociolinguistic and conversation analytic methods was used (Antaki, 2011; Rampton, 2019). Specifically, qualitative data analysis software was used to code and analyze the data in terms of its turn-taking dynamics, topic management, face work and specific aspects of formulation. For the specific focus of the present article, we conducted a complementary analysis using the same software tools to capture transformative sequences in our corpus. More precisely, we scanned relevant previously coded interactional episodes for any manifestations of transformative interaction. While analyzing the ways in which transformative sequences were accomplished in identified episodes, we systematically mapped the affordances (possibilities and constraints) of instrumental awareness raising which is pertinent to the specifically transformative nature of the contact tracing call. Precisely the ascertained tensions informed our analysis written out below. Interactions which exemplify the interactional strategies and identified pitfalls were examined for common or divergent elements, which enabled us to ultimately demonstrate the complexity of attempted transformative work in contact tracing telephone interactions.

## 5. Results and discussion

Our primary goal in this article is to demonstrate the complexity of transformative work in contact tracing calls. We do so by identifying the various tensions and pitfalls which surround the accomplishment of the envisaged cognitive instrumental awareness raising about relevant categories, which is central to the public health task of contact tracing. Specifically, we highlight the following dimensions: (i) how contact tracers deal with the fact that clients may already be quite knowledgeable about what is expected of them in relation to COVID-19 related categories, (ii) what use the contact tracers make of jokes, humorous comments and other mitigating strategies in the delivery of unpleasant messages which come with particular directives which limit behavioral leeway, (iii) what use contact tracers make of formulations which upgrade or downgrade the relevance of particular categorical instructions, and (iv) how contact tracers attend to both anticipated and actual interactional displays of affect and emotion as relevant to their institutional brief of offering support and securing compliance with regulations. In a fifth and final section, we concentrate on how these four dimensions can be discussed together in relation to categorization/particularization work.

### 5.1. Knowledgeable clients

While instructing patients about the public health measures was an essential and obligatory part of the contact tracer’s brief, and hence the script in front of them, in practice, many index patients displayed awareness of the prevention measures, as references to these measures were omnipresent in the media and society at large.^5^ This was especially the case as the pandemic progressed; then, many patients had become quite familiar with concepts that may have been relatively novel to them before Covid. This inevitably intensified by the possibly face-threatening nature of the information exchange, as patients could interpret the repetition of instructions as redundant, or possibly even as insulting or infantilizing. Throughout our corpus, we notice numerous instances in which tracers deal with displays which index knowledgeable clients.

**Table 1:** 

Excerpt 1^6^		
36	CT	now uhm I wanted to share one more thing with you [FIRST NAME IP] now you are a nurse yourself right?
37	IP	. yes
38	CT	yes=
39	IP	=yes
40	CT	now you know that there are uhm isolation measures=and also prevention measures?
41	IP	… (1) yes
42	CT	and you know how the virus works of course right?
43	IP	[yes]
44	CT	. it [survives] on dead surfaces=right like tables cabinets it survives on [glossy surfaces]
45	IP	[yes . yes]
46	CT	screens . right? [tablets]
47	IP	[yes]
48	CT	smartphones *et cetera*= =so then you really should be careful in that regard with your partner . right? . so that you [do] adopt uh
49	IP	[yes]
50	CT	a certain carefulness an&
51	IP	well just now . I have uh have opened everything to air because I have also been told to air often and I have uh . well put all the sheets in the washer and uh . [refreshed everything]
52	CT	no okay [great]

In Excerpt 1, the contact tracer repeats information obtained earlier in turn 36 when referring to the index patient’s professional occupation as a nurse, thus voicing reasonable assumptions regarding the caller’s familiarity with the virus’ inner workings. In addition to employing this strategy to initiate the list of prevention measures, the contact tracer makes a more individualized attempt at securing compliance. Rather than relying on the familiar strategy of listing information, i.e., the strategy commonly used by contact tracers in this part of the scripted conversation, the contact tracer shifts to a question format which implies that the patient is presumably already aware of most of this. Put briefly, the contact tracer draws upon the patient’s perceived status as a knowledgeable client to provide what they presume to be already known instructions about isolation. Note how the tracer’s questions are followed directly by – albeit short – confirmation checks (“right?” in turns 42, 46 and 48), but the tracer does not wait for the patient’s response to these checks. This is noticeable through the amount of overlap in the speakers’ turns. In other words, the tracer does not signal a wish to co-establish information step-by-step together with the index patient, but instead goes through a list of quasi-rhetorical questions in one fell swoop. Perhaps the tracer took the answers to these confirmation checks to be redundant precisely because of the patient’s status as a knowledgeable client. Knowledge display by the client is conversely exemplified in turn 51, when the patient in her turn lists the adoption of very specific prevention measures, to which the tracer responds affirmatively (turn 52, “great”). The details about changing sheets and venting the room in turn 51 equally mark the patient’s keenness to display knowledgeability. There is complexity in the rapport.

Of course, contact tracers will not always be aware of an index patient’s occupational background in health care, especially early on in the interaction. Additionally, even when a contact tracer knows, it will be difficult for them to waive the instruction stage on the basis of patient familiarity with it, because the script demands that tracers provide the same information in every call. An example of such an instance can be found in excerpt 2 below.

**Table 2:** 

Excerpt 2		
26	CT	her . partner your step dad . has he been tested already? or . is that going to happen on the seventh day?=or . how?
27	IP	((adamently)) he only needs to be tested . day seven after xxx the-the index patient has been positive right
28	CT	((hesitantly)) ah yes . uh= =but he also has no symptoms then [at the moment?]
29	IP	[no]
30	CT	no . okay

In this excerpt, the contact tracer is conversing with the index patient’s daughter-in-law, who acts as a spokesperson for the elderly woman who has just tested positive. The daughter-in-law is a general practitioner, and signaled reluctance to cooperate during the call. From the start, she limited her contributions to very short and matter-of-fact replies. In the turns directly preceding the cited extract, the contact tracer mechanically read out loud the list of prevention measures mentioned in the script. This seemed to further annoy the daughter-in-law. In the extract, the tracer quizzes the daughter-in-law to get a better view of the living conditions of the index patient – i.e., who else lives with them, who has already been tested positive, who still needs to be tested, etc. In response, the daughter-in-law corrects her interlocutor (an instance of other-initiated repair with an added qualification; Kendrick, 2017, p. 174: the question about a second test for the index patient’s co-dwellers is received as misleading: “he only needs to be tested on day seven after the index patient has been positive, right”). Turn 27 indexes a well-informed caller who cites the rule which applies to the situation of her in-laws. This case clearly shows how a collaborative rapport between the contact tracer and the index patient may be jeopardized when tracers fail to account for their interlocutors’ pre-existing knowledge. In turn 28, the contact tracer, albeit hesitantly, yields to the authority of the index patient, who, unlike the contact tracer, has been trained medically. The contact tracer’s introduction of yet another detail within a framework of relevant expertise in the second part of turn 28 (“symptoms at this moment”) signals competition.

To prevent friction, such as that illustrated in Excerpt 2 above, some tracers in our corpus hedge their instructions by prefacing them with the observation that the index patient is likely to be a knowledgeable client, as is the case in the opening turn of Excerpt 3.

**Table 3:** 

Excerpt 3		
5	CT	hopefully you are already aware of the necessary prevention measures so that is definitely right because today you did in fact come in as positive which [is why]
6	IP	[yes]
7	CT	that for 7 days … (1) at least you will go into isolation right
8	IP	yes
9	CT	you know this right so definitely stay home so the virus uh diminishes or its spread at least uh stay as far away as possible from your family definitely cover your nose and mouth at all times should you couze=uh cough or sneeze correct use of the bathroom what do we mean by that uh do you live alone or not?
10	IP	… (1) uh yes I live together with my boyfriend but we have two homes so we have just uh [decided]
11	CT	[oh]
12	IP	that I will go to mine and he to his
13	CT	@ there we go that’s great right
[.]		
25	CT	but uh separate uh u-uh use of the toilets is really great and always uh put the lid down disinfect properly always disinfect the surfaces properly and that’s very important=and always proper ventilated air in your home so air properly
26	IP	yes
27	CT	and [obviously]
29	IP	[yes]
30	CT	also keep washing your hands and obviously keep disinfecting right

In this excerpt, the tracer suggests that the patient is probably already aware of some of the government’s recommended prevention measures by expressing the hope that most of the information has already reached the patient. Note that this sequence comes at the very start of the telephone conversation. The tracer’s use of the term “hopefully” (turn 5) can be interpreted as carrying moral overtones. Moreover, the tracer continues the information supply by implying that the index patient will already know what is expected (“you already know this” in turn 9), while nevertheless explicitly going through the listed measures of the contact tracing script. For instance, turn 25 not only details the rule “appropriate use of the toilet,” the contact tracer here also engages in a brief clarification sequence which is first announced (*cf.* “what do we mean by that?,” turn 9) and is then subsequently initiated by a query (“do you live alone, or not?,” turn 9). In the turns that follow, the rule for safe toilet use is applied to the patient’s home context.

The onset of the above sequence is in some respects different from the approach which many contact tracers take. Quite often in our corpus, a contact tracer introduces the long list of measures mentioned in the script by stating the likelihood of the patient’s awareness, adding that the tracer nevertheless “must go through them” for the sake of thoroughness. This is reminiscent of Brown and Levinson’s (1987, pp. 122–125) ‘presupposition manipulations,’ in which speakers invoke presumed shared knowledge with the hearer in an attempt to redress a face threatening act, as in the request: “I know you cannot bear parties, but this one will be really good – do come!.” However, in Excerpt 3, the stance is somewhat different, precisely because of the arguably moralizing replacement of ‘likely’ or ‘probably’ by ‘hopefully’ in turn 5. One can imagine an index patient who is not that in tune with the Covid-19 measures to be potentially insulted by the strategic appeal to pre-existing knowledge. In other words, interactional strategies of positive redress may backfire, and an assumption of ‘likely’ familiarity is probably less risky than one of an expressed ‘hope’ of familiarity.

### 5.2. Upgrading and downgrading formulations

While the display of sensitivity to the patient’s knowledge status provides one way of dealing with clients one has not met before, which may contribute to successful awareness raising, contact tracers may also seek to secure compliance by upgrading (and in some cases, downgrading) the relevance or importance of specific prevention measures (Bilmes, 2018). Excerpt 4 illustrates how the specific rule “wear a face mask” is nuanced and negotiated interactionally.

**Table 4:** 

Excerpt 4		
269	CT	… (0.8) uh so [for]
270	IP	[yes]
271	CT	you yourself it really is important that you uh remain careful if you are close to him do you wear a mask when you are close=[close]
272	IP	[yes]
273	CT	to him in the same room or?
274	IP	… (2) ((hesitantly)) uh I have not when we-we do when we are taking care of him but=but when we are just in the same room then not
275	CT	no? ok but you do keep enough distance then?
276	IP	… (1.8) yea w-well yes of course
277	CT	yes well uh do in fact try to keep that distance as much as possible wear a mask when you have to be in close proximity to your husband uh additionally make sure to regularly uh ventilate the room right= if you are in the same room with distance but y- if you keep the windows shut then the virus can actually start to expand so it really is important to ventilate enough so those virus particles are uh well yea ventilated

When the contact tracer asks the index patient (in turn 271) whether she wears a protective mask when in the same room as her partner, who was recovering from surgery, the index patient initially responds hesitantly, and reports that this varies: she wears a mask when she takes care of her partner, but not when they are simply together in the same room. In response, the contact tracer seeks further clarification (turns 273 and 275), apparently preparing the ground for a justification of the index patient’s reported tendency to not always wear a mask. The contact tracer provides context-sensitive advice, which is tailored to the index patient’s specific situation, and, in doing so, downgrades the public health appeal to constantly wear a mask indoors to prevent further spread of the virus when in the co-presence of a fellow dweller. Instead of affirming the rule, the contact tracer focuses on the complementary advice of ensuring proper ventilation of the room, which is not a prevention measure that is mentioned explicitly in the contact tracing script.

A contact tracer may also upgrade the gravity of the situation, as exemplified in Excerpt 5 below. Immediately before this excerpt, the index patient asked the contact tracer why he is being called a second time. The tracer briefly explains that new case files are now made for each member of the family, and stresses how important it is that the index patient cooperates.

**Table 5:** 

Excerpt 5		
138	CT	=that’s why you uh have been contacted these past few days . right to take a [look at]
139	IP	[yes]
140	CT	uh to protect the people around you . right protect [your family=]
141	IP	[yes=]
142	CT	=protect your co-dwellers since uh. yea it’s a disease= it’s really not something to laugh at mister=uh [FIRST NAME] it’s uh very uh
143	IP	[yes ok]
144	CT	[very] dangerous of course right we really should uh [instill]
145	IP	[yes]
146	CT	some carefulness right that’s why we do this . right this is purely for your [safety]
147	IP	[xxx]
148	CT	for your protection too right
149	IP	okay

In a series of categorical classifications (“it’s a disease” in turn 142, “very dangerous of course” in turn 144, “purely for your safety” in turn 146, “your protection too” in turn 148), the contact tracer in this excerpt scales up the risk associated with a Covid infection. Quite likely, this forms part of an attempt to secure compliance with the measures that apply. Notice the additional references to responsibility for the “the people around you” (turn 140) and “your co-dwellers” (turn 142), as well as the use of a formulation which underlines the seriousness of the situation: “it’s not something to laugh at” (turn 142).

### 5.3. Joking and the use of humor

In addition to displaying sensitivity to patient knowledge and the calibration of formulations (up- or downscaled), contact tracers often draw upon the use of humor to alleviate the inevitably face-threatening character of the contact tracing call. Humor can aid the contact tracer in diffusing certain perceived or anticipated tensions. It can also backfire and result in friction between the tracer and the patient.

In Excerpt 6 below, the contact tracer relies on a joking move to mitigate certain sensitive questions about the patient’s private living conditions. The relevance of this in terms of awareness raising is that this bears on the application of the category of ‘self-isolation’, that is, the extent to which the patient can effectively self-isolate at home.

**Table 6:** 

Excerpt 6		
52	CT	[yes] exactly right … (1) uhm . now it is the case that the-the chance is . well the-the-what is being asked is definitely to stay as far away from your family as possible right . [uhm but it’s] difficult of course
53	IP	[yes but . the&]
54	CT	you uh-probably do not live in a villa where everyone has a separate wing . right? @@
55	IP	no . @ unfortunately not . [no]
56	CT	[yes] most people I talk to . do not either= =they really do not . believe [me]
57	IP	[yes] I would think so
58	CT	but it’s definitely very difficult and if you indeed cannot . uh-go in self-isolation . uh … (1) [and definitely=]
60	IP	[yes=]
61	CT	=also with-how old are the kids by the way?

Following an upgraded formulation of the recommended measure (i.e., “definitely to stay as far away from your family as possible,” in turn 52), the contact tracer expands on the application of the rule with a pessimistic assessment of the size of the patient’s home. Humor resides in the exaggerated comparison (in turn 54) with the ‘COVID-19 optimal’ condition of a house in which each dweller occupies a separate wing: “you probably do not live in a villa where everyone has a separate wing.” As can be seen in the excerpt, the contact tracer’s joke is appreciated by the patient, who replies with laughter and responds jokingly with regrettable agreement (“unfortunately not no,” turn 55). Interestingly, even though the patient’s positive response suggests affiliation and hence a successfully accomplished joke, the contact tracer nevertheless continues with an explicit justification of the patient’s answer; the contact tracer reports that most people who are contacted do not live like that, and that self-isolation is a challenge (turns 56 and 58). In doing so, the tracer downsizes the effect of the joke, which was – perhaps somewhat ironically – already an attempt to mitigate the tracer’s initial question regarding isolation. Humor is risky, and a Covid-infection is a serious matter. A patient could take offense at the joke, which is precisely what contact tracers may want to avoid in the first place. The transition from the joking turn pair to its justification marks a shift in tone: now the contact tracer is being serious and the topic shift in turn 61 functions as a clear segue into the next item of the script.

The contact tracer in Excerpt 7 below also uses humor to mitigate the instructions about isolation, compared to quarantine. The joking turn occurs in turn 628. The index patient reciprocates.

**Table 7:** 

Excerpt 7		
617	IP	@ so my husband is positive he has to go in isolation [right]?
618	CT	[yes] that’s right and you [have]
619	IP	[yes]
620	CT	not yet [tested]
621	IP	[I]
622	CT	positive so you are just in quarantine so you can still complete essential activities . should [you still test positive]
623	IP	[ah so I can]
624	CT	tonight then you also have to go into isolation which means that you also you cannot go out anymore and that someone else will have to get groceries and that’s how it works [actually]
625	IP	[okay so this afternoon] I can still&
626	CT	yes
627	IP	get groceries in a safe [way]
628	Ct	[yes] you can still go to the bakery=you can to the butcher . uh if you have torn pants then you are also allowed to get a new pair of pants now of course we do ask people to avoid this because I think you @ still have @ enough @ pants @ in your wardrobe @
629	IP	@ @
630	CT	to survive @ @
631	IP	@ @ @

The joke is that the patient can still go out to replace an imagined pair of torn trousers. In passing, note what is perhaps an unexpected formulation in turns 622–624: while awaiting a formal test result, the patient can go out to do shopping. This is a rather unusual piece of advice, as most other tracers in our corpus would urge citizens to stay inside while awaiting their formal test results. The “stay-at-home”-rule is being downgraded (see section 5.2 above). In any case, Excerpt 7 shows how, in a contact tracing call, humor can be used as a dynamic strategy, in this case to mitigate the imposition of an instruction. Humor, it is hoped, contributes to securing compliance.

A similar example can be found in Excerpt 8.

**Table 8:** 

Excerpt 8		
286	CT	[yes] no exactly as soon as we have one uh symptom right like coughing a throat ache uh a sore feeling in the throat then a test really is [in order]
287	IP	[I did in fact] have
288	CT	yea no it’s [really quite uh]
289	IP	[yea . yeaa] yes well I=will=tell=him-I=will=tell yes ok and then I have to @ go into a room and they have to bring my food- like they have to take care of me then right and I can only use one- like I can only use my own toilet [and uh . am I also allowed to]
290	CT	[yes they are obliged to treat you] they really are obliged to treat you like a princess right so that’s really mandatory uh @ no but uhm [it really is uh]&
291	IP	[yes yea yea]
292	CT	the idea is [that they bring]&
293	IP	[@]
294	CT	food *et cetera* yes so uhm you can also see it as a positive thing right
295	IP	yes ok yes exactly I will let them spoil me
296	CT	[yes like that @]

In Excerpt 8, the patient demonstrates her knowledge of the instructions (*cf.* “then I have to go into a room …”). Compliance is secured and arguably sealed with the contact tracer’s humorous comment: “they are obliged to treat you like a princess, right” (turn 290). The humor not only serves to soften the blow of the index patient’s isolation, but also reformulates an imposition (*cf.* the infected patient should not be doing things around the house) metaphorically and positively in terms of ‘treating someone like a princess’. Notice especially the joking, yet slightly risky formulation “they are obliged to” (turn 290). In this case, the index patient again appreciates the joke. Following the positive reception (laughter in turn 293), the tracer adds on to the humoristic comment, prompting the index patient to express agreement with the advice (“yes exactly”) and adopt the contact tracer’s suggested stance (“I will let them spoil me”). Although in this particular situation the joking has resulted in a display of reciprocity, one can certainly imagine how the humor could backfire as the tracer’s joke for instance implies that the patient is otherwise ‘not treated as a princess.’ The use of humor as a mitigation strategy arguably is a delicate matter.

Humor also occurs as a mitigation strategy in the stages of the call where information is being obtained from the index patient. This can result in a relatively intrusive experience for index patients. Interestingly, responses which suggest failed mitigation tend to occur most of all when humor is directed at the person rather than situational humor. Excerpt 9 exemplifies the former.

**Table 9:** 

Excerpt 9		
292	IP	xxx ((they were just now?)) it’s all the same he said
293	CT	is it all the same are you sure? yea?
294	IP	well yea so well yes=yes=yes well yea xxx ((because we have already been))
295	CT	@@@ you are twins in all respects really&
296	IP	well yea
297	CT	even that is the same @@@
298	IP	y-yea that-that is all-all the same=
299	CT	=all the same your brother has not seen anyone else?
300	IP	. no . well no because we are always xxx ((together / but here))

Here, the contact tracer is discussing the contacts of the index patient’s twin brother. In turn 293, the contact tracer seeks additional confirmation for the information shared in the previous turn. In doing so, a potential face threat is manifested in the specific formulation of this question for confirmation: “is it all the same/are you sure?” (turn 293). At this point, the contact tracer makes a more person-directed joke, laughingly stating that the index patient and his brother are “twins in all respects really” (turn 295). Even though humor, and shared laughter specifically, can indeed be a valuable tool for generating and ensuring rapport between interlocutors (Jefferson et al., 1987), without a laughing ‘second’ as a response, this mitigation strategy can be considered unsuccessful (Jørgensen, 2019). The contact tracer’s attempted repetition of the joke in turn 297 offers the index patient another opportunity to show appreciation of the joke (Jørgensen, 2019, p. 391). The attempt fails (there is no laughter from the index patient). This again shows how the use of humor is not without risk. While it can take on a valuable transformative function and alleviate friction or predicted ill-perception, when it is not well-received, humor may end up compromising or threatening rapport. See Haakana (2001) on recipient-laughter as a troubles-resistive resource and Jefferson (1994) on failure to laugh as indexing troubles-receptiveness.

### 5.4. Expressions of affect and emotional displays

Throughout the contact tracing interactions, tracers are faced with index patients who voice personal concerns and respond emotionally. Although the contact tracers were strongly reminded by the Flemish Agency of Health and Care that the contact tracing call must be a care-centered conversation, they received limited training on how to respond empathetically and attend to expressions of emotion or affect. Consequently, we observed significant variation in how tracers attended to care-centered concerns during the contact tracing interactions. Our interest in this section is in how patient-initiated displays of affect and emotion are responded to, organized as their expression is, in interactional sequences. It may prove difficult for a contact tracer to secure compliance of an emotionally distressed patient (note equally how an orientation to institutional tasks constrains the display, recognition and validation of clients’ emotion displays; Jørgensen, 2019).^7^ In other words, the transformative goal of a contact tracing call may in some cases require explicit attention to the worries and concerns which are voiced by the index patient, inviting on-record validations of distress and upset (compare with Muntigl et al., 2017, 2023 on comparable interactional work in psychotherapy).

In Excerpt 10 below, the contact tracer engages directly with the caller’s successive expressions of affect.

**Table 10:** 

Excerpt 10		
62	IP	I really am kinda [paranoid] in that regard @
63	CT	[uhu] no okay . but do not = have = to = be so really paranoid but . keep the inner workings of the virus in mind right?
64	IP	yes
65	CT	uh [and then] work like that really right?
66	IP	[yes]
67	CT	uh=
68	IP	=yea uh even when I cook I always=wash my hands=and stuff because uh my colleagues said it themselves it’s surprising that = that you got it@ because I do not know a- nobody who sticks to the rules as well as you do so I do find that a bit unfortunate
69	CT	nyea [e&]
70	IP	I did manage to last a while
71	CT	yes exactly [uh]
72	IP	[for uh]
73	CT	I was recently talking to a couple they had been [uh] locked up since [MONTH]
74	IP	[((coughs))]
75	CT	right? since uh [MONTH] @ so that was already uh-about 8 months right? and eventually they got it as well because of a short contact with of about half a minute so uhm . uh so you really do not have to feel guilty because of it=right [FIRST NAME IP] [uhm no]
76	IP	[yea that was] bit uh but yea uh- you cannot stop it=right it’s so strong I mean&
77	CT	no . exactly . what we can do is act the best way we can . right? [and uh]
78	IP	[yes]
79	CT	protect those that are of course closest to us right?

The index patient’s admission of being “paranoid” about it all is responded to as unnecessary. The patient continues to voice affect: colleagues are quoted as being surprised that someone who took so much care in following the rules nevertheless got infected. The contact tracer responds to this with a ‘second story’ (Arminen, 2004), which echoes the symbolic significance of the index patient’s brief narrative. It is about a couple who voluntarily quarantined but nevertheless contracted Covid. Echoing the caller’s experience, the sequence is rounded up with an advice not to feel too guilty about contracting the virus. In this excerpt, the contact tracer’s response to the successive expressions of affect disaffiliatively plays down the feeling, putting in the foreground instead the instrumental focus of the call: patients are advised to be aware of the virus’ inner workings (turn 63), and an expression of disappointment at being infected is turned into a motivating conclusion with a moral angle: as the virus is so strong and cannot be stopped, we must “act the best way we can” (turn 77) and “protect those that are of course closest to us” (turn 79).

In a few rare instances, the index patient’s voiced distress about their recent plight caused the conversation to drift away from the topical priorities of the contact tracing script. The index patient in Excerpt 11 below, an elderly woman, derails the conversational task by telling the tracer how she had recently lost her husband and was in an unfortunate feud with her neighbors because they had damaged a part of her home during renovation works. The tracer pauses the script and allows the patient to tell her story and voice her hardship in detail.

**Table 11:** 

Excerpt 11		
108	IP	I am having [some] difficulties at the moment ((voice cracks))
109	CT	[yes] yes I can really understand that [madam]
110	IP	[yes uh] it’s that whole situation
111	CT	yes=
112	IP	=yes . yes
113	CT	yes I’m uh-I’m sorry to hear that uh that your neighbor is acting [in such a way]
114	IP	[yes such] educated people . [a lawyer]
115	CT	[yes] yes [exactly]
116	IP	[yes the-] there was someone who said . you know about lawyers they think they have got=it [all]
117	CT	[yes]
118	IP	uh yes
119	CT	yes
120	IP	yes . and yet there’s an insurance oh God . uh yes but yes the insurance will now try to continue yes I do not know the result yet
121	CT	nyea
122	IP	uh yes . it’s not the end of the world no ((voice cracks))
123	CT	no but it’s not [pleasant right] . no
124	IP	[oh well]
125	CT	no
126	IP	yes madam yes . look
127	CT	yes
128	IP	uh
129	CT	the best you can do is continue slowly step by step madam [and uh make sure that uh&]
130	IP	[well yea that’s uh that’s right but it’s] sometimes- It’s [sometimes difficult] why
131	CT	[it’s difficult. yes]
132	IP	do you know when I feel best when I’m outside I=I- I can see the birds here [yes] I live by the forest
133	CT	[yes]
134	IP	. the birds here oh yes . in the=garden my chores in the garden but yes now there is not much work in [the garden right]
135	CT	[no no]
136	IP	I do have the moments- ((xxx))
137	CT	yes = yes
138	IP	well yes
139	CT	yes
140	IP	oh [how] one . can suffer
141	CT	[mhm] nyea . nyea true . unfortunately well [but uh]
142	IP	[yea right] yes . and none of it is necessary
143	CT	no . but still it happens

The excerpt quotes only a limited chunk of the topical digression. The sequence lasted from turn 36 until 224, and the themes resurfaced from turn 280 until 450. In other words, more than half of this 35-min conversation was spent on displays of support in response to the patient’s affective expressions of distress. In addition to the details of hardship, the patient also reports on moments of consolation (turns 132 and 134). The tracer responds empathetically, by endorsing the client’s voiced affect (e.g., turn 123 and turn 143), offering affiliative receipts through backchannel signaling a listening stance (e.g., turn 115), and expressing regret at misfortune (e.g., turn 113). Throughout this excerpt, and indeed most of the interaction, the tracer maintains conversational space for the index patient to engage in life-story telling. The exchange is fairly unique in our corpus, as virtually all contact tracing calls in our corpus adhered far more closely to the tracers’ script.

Beck & Ragan (1992) note how most institutional interactions come with leeway toward chat taking place alongside a focus on institutional task and purpose; such marks the empathic integration of interpersonal and task-focused dimensions in institutional encounters. In turn 123, the tracer explicitly adopts the index patient’s perspective. The turn marks an undoubtable shift from an information-centered contact tracing call to client distress-centered talk. The contact tracer’s response of shared affect in this turn comes at a point where the index patient minimizes her own complaints (“it’s not the end of the world no”) and then her voice cracks, in a (possibly involuntary) display of distress. Throughout the remainder of the conversation, the tracer repeatedly voices pieces of advice as a form of emotional support, this way allowing and arguably even encouraging the woman to continue her personal story. In other words, the contact tracing frame appears to have been temporarily transformed, as an additional layer of supportive chat in response to troubles talk is ‘keyed’ on top of the frame of the contact tracing interaction (Goffman, 1974, p. 40ff).

Does this exchange come close to therapeutic counseling? Across the corpus, we have noted the contact tracer’s use of distress recognition turns, expressions of shared affect and non-specific supportive moves (e.g., turn 129 in Excerpt 11). We did not note any topicalizing responses which echo the strategies of emotion-focused therapy such as immediacy questions or modulating directives (Muntigl et al., 2023). Interestingly, *take-your-time* responses were only noted when index patients failed to come up with particular bits of information, but not when they displayed distress.

As was illustrated by the previous two excerpts, patient-centered support was an important aspect of the contact tracing conversation, but this is not to say that our corpus does not contain any instances in which the importance of a caring and empathetic stance is disregarded by the tracer. An example can be found in Excerpt 12.

**Table 12:** 

Excerpt 12		
		and have you been anywhere Saturday morning?
94	I	(3) uhm . no
95	CT	. afternoon evening neither? for example to a&
96	IP	uh no then someone was here who turned out to be infected but I was here= =so someone ran into my car in front of my home
97	CT	okay
98	IP	so . but yea that contact was all outside from a distance with the person who-who caused the accident with the police there so . that was all outside and from a distance
99	CT	all from a distance . okay right . ok th- I’ll return to the contacts later so yesterday you were . for half an hour . at work you said
100	IP	yes
101	CT	you work at an education-just at a school
102	IP	yes
103	CT	(3) okay let me see
104	IP	but the CLB is already handling that right= xxx (so to uhm) to check . the risks there
105	CT	okay alright (4) and you also did not . go to a party for example or to an event . by a sports organization
106	IP	no I did go to my parents= =as I said previously
107	CT	okay
108	IP	(5) parties aren’t allowed by the way
109	CT	(2) yea now that everything has everything has been eased up uh . it could still happen
110	IP	[yes . yes]
111	CT	[I’m only asking] just to be [sure]
112	IP	[I have] an entire year . incredibly careful because I’m a high-risk patient
113	CT	okay but that’s great
114	IP	((sighs)) so-so- (2) and now this weekend I saw a few more people because of that accident and because of . Mother’s Day
115	CT	((repeats silently)) because of Mother’s Day

In Excerpt 12, the contact tracer is in the process of asking the index patient about her contacts in the days leading up to the positive test result. Directly preceding the excerpt, the index patient informed the tracer that she had briefly seen her parents on Sunday to celebrate Mother’s Day. In the cited excerpt, the tracer continues by asking the index patient if she has also seen anyone the day before, on Saturday. The patient answers that she did and elaborates on the unusual circumstances that caused the contact: a stranger had run into her car while it was parked on the driveway; as a result, the patient had brief contact with the driver and an unspecified number of police officers, one of whom later turned to have been infected with COVID-19. The caller ends this brief recount by mentioning how this interaction took place outside and at a safe distance from the other individuals.

In the subsequent turns, the tracer does not acknowledge the unfortunate event of the accident, and only summarizes the information that pertains directly to the contact tracing script, i.e., “all from a distance ok” (turn 99). The tracer announces a further return to “the contacts” in turn 99, and moves to the next item in the script, *viz.* the index patient’s profession. The patient assures the tracer that the school’s CLB – a Flemish educational support organization – has already been informed and will be taking the necessary precautions. This information prompts the tracer to proceed to the next item in the script and ask the patient if she has attended any other gatherings or parties. The patient replies in a frustrated tone, stressing that she had already mentioned the meeting with her parents (turn 106), and subsequently reminds the tracer of her awareness that “parties aren’t allowed by the way” (turn 108). The caller goes on to highlight how she is a high-risk patient and had been very careful throughout the entire year, but that this weekend was exceptional because of Mother’s Day and the accident. Note how the tracer simply repeats the former (“because of Mother’s Day,” turn 115), while writing down the information.

Excerpt 12 is a telling example of how contact tracers may in some cases fail to respond empathetically during contact tracing interactions. In this specific example, the tracer does not invite the index patient to elaborate on the accident that presumably led to the patient’s COVID-19 infection (turn 96). In fact, the accident itself is never explicitly acknowledged by the tracer, nor is the additional emotional impact of a positive test result. This is exacerbated in the final turns of the excerpt, when the index patient, who is audibly frustrated with the tracer, mentions she is a high-risk patient who had been avoiding regular contact for over a year and that the car accident was unfortunately one of the reasons she had seen more people during the weekend. Again, the tracer does not acknowledge the accident. Viewed from the point of the institutional task, this is irrelevant information. Nor does the contact tracer ask for clarification about the caller’s status as a high-risk patient, while this would constitute a factor which warrants customized advice.

### 5.5. General rules and particularized advice

The interactional orientation of the instruction and advice stages of the contact tracing call can be identified as oriented to heightened awareness about the nature and scope of behavior-relevant categories such as ‘isolation,’ ‘quarantine,’ etc. for which general rules applied throughout the period in which contact tracing was conducted. In partial contrast with the non-person specific, across-the-board application of rules and measures, a considerable amount of interactional time appears to be invested in the assessment of how the categories apply to the individual caller, how they require translation to local circumstances, and in some cases, intensification, modification, even exception vis-à-vis the specific situation of the index patient. The field of play is that of real, envisaged, and desired behaviors in response to the conditions of the COVID-19 pandemic. Following Billig’s (1985) seminal article on categorization work, the contact tracer can thus be viewed as engaged both in fitting realities into categories, i.e., categorization work, as well as adjusting individuals to the application of categories, in other words: particularization work, which, in some cases, renders categories malleable. As discussed in detail in Hall et al. (2006, p. 27), Billig’s work emphasizes how the negotiation over the characteristic features of specific categories is often a matter of situational application and/or a source of argument and debate. Assigning an entity or instance to a category requires a formulation which can be both supported and challenged by specific circumstances.

Applied to the context of the contact tracing call, categorization and particularization work will be intimately related since it is primarily by investigating the relationship between general measure and the case of the index patient, that categories such as quarantine or isolation can be rendered meaningful and consequential in interaction. In the course of this, a range of interactional moves and strategies come into play such as: claim authority about a category, attend to the patient’s affective response, appeal to responsibility or moral duty to observe a measure, work up the relevance of particular features, upgrade or downgrade a measure by rendering it in categorical terms or relaxing its importance, joke about the category, etc. Active categorization/particularization work is likely to be fundamental to a client’s awareness about and acceptance of a measure or rule, including the action imperatives which it entails (Mäkitalo, 2014).

One brief example here, which quotes a short sequence from one call, underlines the contact tracer’s active work of categorization. Tying together the observations made in the different results sections, in this instance, the contact tracer avails himself of an upgraded formulation, a particularization and face-redressive appeal to the brevity of the measure.

**Table 13:** 

Excerpt 13		
84	IP	uhm . so me- alone in a room and they can-cannot leave our home?
85	CT	yes so theoretically if you live in uh an apartment theoretically the husband and the children can just walk around in the apartment but you really have to be separate . stay in a separate room
86	IP	mhm
87	CT	also sleeping by yourself ideally separate at least because I personally find one and a half meters too short three w-three @ I always say three stay three meters apart from each other [or]
88	IP	[yes]
89	CT	but just power through for a bit then it’ll hopefully be over soon

In the exchange, the measure of recommended self-isolation is being worked up interactionally, while being applied to the specific situation of the index patient. We note the use of a core formulation (turns 84–85: in principle, no one can leave the dwelling; turn 85: the index patient is alone in a separate room). Detailed qualifications are added for physical distance (turn 87: there is upgrading in the insistence on 3 meters physical distance, instead of the standard publicly recommended one and a half). The contact tracer adds a particular distribution of roles as to who can walk around freely and who needs to secure the distance (infected patient: “in a separate room” vs. the others: “just walk around in the apartment”). The instructions are couched from the dwellers’ perspective as unpleasant but necessary (“power through for a bit”) and, further minimizing the imposition on the dwellers’ freedom of movement, as an uncomfortable situation which hopefully will not last long (“hopefully be over soon”).

Active categorization work during interaction is arguably conditional for the accomplishment of raised awareness about categories of social reality and their acceptance in particular terms. Active categorization work equally invites attention to the various tensions and pitfalls which surround the successful interactional accomplishment of transformative sequences of talk.

## 6. Conclusion

In this paper, we have shed light on the characterization of COVID-19 telephone contact tracing in terms of doing ‘transformative work.’ Our analysis of the recorded conversational sequences has laid bare the complexity of this kind of work by highlighting some of the inevitable interactional challenges which occupational contact tracers face in the institutional accomplishment of the envisaged outcome of raising index patients’ instrumental awareness which is key to the task of contact tracing. Particularization work turns out to be central to this. This aligns with the idea of a contact tracer as an initiator who is professionally required to interact responsively in the telephone contact with index patients. Specifically, we have addressed the dimensions of dealing with clients who are already quite knowledgeable about the envisaged outcomes of the contact tracing call, the use of humor and other mitigating strategies in the delivery of unpleasant behavioral directives, as well as the specific use of formulations which up/downgrade the relevance of instructions – and, finally, the paradoxes which surround client-initiated displays of emotion and affect. The contact tracers’ professional-occupational engagement with the interactional contingencies of displays of affect and distress hints mostly at the use of more general empathetic response turns, while underlining the potential tension between experience sharing and the effective pursuit of instrumental goals, in this case: getting people to behave in a way which is instrumental to containing the spread of COVID-19.

Interactional analysis of telephone tracing practice is relevant for institutional practice. It can contribute to an enhanced understanding of how the institutional work is actually being accomplished in moment-to-moment sequences of talk. In this way, the perspective on its *ongoing*-ness and its susceptibility to the ‘local conditions’ of dealing with real clients and their specific situations can become a useful resource for fostering reflexive practice and professional-occupational self-awareness. Reflexive analysis which highlights toolkit adaptivity in accordance with local interactional affordances are undoubtedly useful in a framework for training which goes beyond the instillment of particular communicative values and the prescriptions of a particular preferred script.

## 7. Transcription conventions

The following conventions were used when transcribing the data reported on in this chapter (*cf.* Du Bois, 1991):

.   short pause.
… (0)   long pause expressed in seconds (starting from 1″).
[xxx]   overlap.
((xxx))   interpretative comment
&   interruption.
=   latching.
@   laughing.
-   self-repair.
?   rising intonation.
xxx   unintelligible.

## Data availability statement

The datasets presented in this article are not readily available because requests to access the datasets should be directed to Stef.Slembrouck@UGent.be.

## Ethics statement

The studies involving human participants were reviewed and approved by (1) for Ghent University, Ethics Committee, Faculty of Arts & Philosophy, (2) for the University of Antwerp, Ethics Committee, EASHW (Ethical Advice Social & Human Sciences). Oral informed consent for participation was obtained. Written informed consent was not required for this study in accordance with the national legislation and the institutional requirements. Consent was obtained from the individual(s) for the publication of any potentially identifiable images or data included in this article.

## Author contributions

SS was the PI of the UGhent leg of the project, acted as first author, drafted the manuscript while assuming final responsibility for the paper. MV was the PI of the UAntwerp leg of the project, acted as second author in a detailed feedback role. RDT was employed by the project as a research officer, drafted the initial excerpt analyses, and provided the data translations. ASB was employed by the project as a research officer. SVDG was briefly employed by the project as a research officer and conducted the interview analyses in the first stage of the project. SS, MV, RDT, ASB, and SVDG contributed to the conceptual planning of the manuscript and the motivated selection of cited data fragments was done as a team. All authors contributed to the article and approved the submitted version.

## Funding

This study was carried out through Project Grant G0G6120N of the Flemish Science Foundation (‘Fonds voor Wetenschappelijk Onderzoek, Vlaanderen’). The project’s title was “Effective information exchange and care orientation in COVID-19-related contact tracing phone calls. An applied sociolinguistic and conversation analytic enquiry into optimizing interactional dynamics and pragmatic awareness”. The 2020 project funding line Covid-19 (2nd call) could only be applied for in the form of jointly-held inter-university projects. The Ghent University part amounted to € 117.500; for the University of Antwerp, this was € 114.700.

## Conflict of interest

The authors declare that the research was conducted in the absence of any commercial or financial relationships that could be construed as a potential conflict of interest.

## Publisher’s note

All claims expressed in this article are solely those of the authors and do not necessarily represent those of their affiliated organizations, or those of the publisher, the editors and the reviewers. Any product that may be evaluated in this article, or claim that may be made by its manufacturer, is not guaranteed or endorsed by the publisher.

## Footnotes

^1^See https://www.who.int/news-room/questions-and-answers/item/contact-tracing.

^2^E.g., a collective lockdown.

^3^Measures and advice evolved over time. E.g., the first lockdown had stages where walking in public parks was forbidden, while the second lockdown allowed people to meet and move around in ‘safe bubbles’ in open air. At one point in the first lockdown, advice was added about necessary distances while running or cycling when sporting outside. This was later retracted. Similarly, in the course of 2022, the importance of infection by touch of spoiled objects was toned down, while the avoidance of airborne transmission and the need for ventilation was absolutely prioritized.

^4^The project’s title is “Effective information exchange and care orientation in COVID-19-related contact tracing phone calls. An applied sociolinguistic and conversation analytic enquiry into optimizing interactional dynamics and pragmatic awareness” (Project number G0G6120N). All data were securely stored and processed in line with ethical and GDPR-related guidelines.

^5^It must be noted that public information campaigns were not restricted to media channels. Included must be warning signs and posters in public places, stickers on the ground to manage the circulation of people, stewards in busy places, outreach by civil society intermediaries to inform hard-to-reach populations, etc.

^6^See section 7 for the transcription conventions used in the data excerpts. All cited excerpts concern contact tracing calls that were conducted in Dutch. The transcribed excerpts are quoted in English translation.

^7^Note in passing that the connection between various emotions and compliance with safety measures is subject to variation. With specific reference to the COVID-19 pandemic, Guo et al. (2023) report in their cross-national study (China, Germany, USA) how increased panic, anxiety and sadness tend to lead to higher compliance, while rising anger, loneliness, and impatience decrease compliance levels. However, as mentioned earlier, our primary interest here is not in mental states, but in the sequential dynamics of emotional display (Goodwin and Goodwin, 2000).
